# Interaction of Energy Drinks with Prescription Medication and Drugs of Abuse

**DOI:** 10.3390/pharmaceutics13101532

**Published:** 2021-09-22

**Authors:** Olga Hladun, Esther Papaseit, Soraya Martín, Ana Maria Barriocanal, Lourdes Poyatos, Magí Farré, Clara Pérez-Mañá

**Affiliations:** 1 Clinical Pharmacology Unit, Hospital Universitari Germans Trias i Pujol, Institut de Recerca Germans Trias i Pujol (HUGTiP-IGTP), 08916 Badalona, Spain; ohladun.germanstrias@gencat.cat (O.H.); smartins.mn.ics@gencat.cat (S.M.); abarrio.germanstrias@gencat.cat (A.M.B.); lpoyatos@igtp.cat (L.P.); mfarre.germanstrias@gencat.cat (M.F.); cperezm.mn.ics@gencat.cat (C.P.-M.); 2 Department of Pharmacology, Therapeutics and Toxicology, Universitat Autònoma de Barcelona, 08193 Cerdanyola del Vallés, Spain

**Keywords:** energy drinks, caffeine, drug interactions, pharmacokinetics

## Abstract

In recent years, the consumption of energy drinks (EDs) has become increasingly popular, especially among adolescents. Caffeine, a psychostimulant, is the main compound of EDs which also contain other substances with pharmacological effects. This review aims to compile current evidence concerning the potential interactions between EDs, medicines, and drugs of abuse as they are frequently consumed in combination. The substances involved are mainly substrates, inductors or inhibitors of CYP1A2, psychostimulants, alcohol and other depressant drugs. Furthermore, intoxications reported with EDs and other substances have also been screened to describe acute toxicity. The results of our review show that the consumption of both EDs alone and in combination is not as safe as previously thought. Health professionals and consumers need to be aware of the potential interactions of these drinks as well as the absence of long-term safety data.

## 1. Introduction

Since the 1980s, the consumption of energy drinks (EDs) has increased, with teenagers and young adults representing the principal consumers [1]. In a United States national survey [2], the greatest prevalence of ED consumption was found among 13–24 years old (10% of caffeine consumers). In a European one, 13% of daily caffeine intake in adolescents was from EDs [3]. Combining EDs with alcohol is also popular among young people, either as a mixed cocktail or in the same drinking session [4]. For instance, one in four youths in North America had consumed alcohol with EDs during the previous year [5,6]. In Europe, 48% of university students using EDs had taken them with alcohol [7] while in Australia half of respondents reported mixing alcohol and EDs at least once in the last year [8]. In the 2019 survey of Monitoring for the Future, between 10% and 12% of students in 8th, 10th, and 12th grades reported using one or more EDs per day in the United States. In the same report, 4% to 5% of students in all three grades reported use of one or more energy shots per day, each typically coming in containers of two to three ounces [9].

The latest Survey on Drug Use in Secondary Schools in Spain (ESTUDES survey), designed to collect data on situations and trends in drug use and other addictions among secondary school students aged 14 to 18 years, reported that 4 out of 10 had drunk EDs in the previous 30 days. Prevalence was found to be higher in boys (49.7%) than girls (31.1%). Regarding consumption of EDs with recreational drugs [10], alcohol was the first concomitant drug (74.4%), followed by tobacco (40%), cannabis (31.3%), sedatives (8.3%), cocaine (1.85%), and ecstasy (1.3%) [11].

The main ingredient in EDs is caffeine. Depending on the brand, other components include guarana (a plant containing concentrated caffeine), taurine, ginseng, *Ginkgo biloba*, 5-hydroxytryptophan, bitter orange, B vitamins, yerba mate, glucuronolactone, and high sugar content [12,13]. The short and long-term health effects of many of these ingredients, and ED consumption in general, are, for the most part unknown [13].

Young people report ED consumption as conferring increased energy, augmented alertness, and improved athletic performance [14,15]. In recent years, many authors have observed that ED intake causes adverse effects, including high blood pressure, changes in corrected QT interval (QTc), serious cardiovascular events, digestive and kidney disorders, metabolic adverse effects, poor sleep, neuropsychiatric adverse effects, seizures, and death [16,17,18,19,20,21]. Furthermore, several studies suggest that EDs may serve as a gateway to other forms of substance dependence [16,22,23]. Additionally, an association with suicide plan/attempt in a paediatric population with a frequent use of EDs (≥5 times/week) has been described [24].

The purpose of this narrative review is to outline the potential interactions of EDs with medication and drugs of abuse, and perform a literature search of reports of intoxication, even those with fatal consequences.

## 2. Materials and Methods

We carried out the search for interactions and cases of intoxication with EDs on the PubMed/MEDLINE and Cochrane Library electronic databases. The keywords employed were: energy drink combined with interaction, intoxication, poisoning, emergency, fatal outcome, adverse event, death, alcohol interaction, methanol interaction, cocaine, amphetamine, methamphetamine, mephedrone, nicotine, ecstasy, MDMA, heroin, marihuana, cannabis, medical drugs and herbal medicine. Interactions mentioned in reference books and online databases [25], and in the product information of caffeine-containing medication [26], were also included.

Additionally, we looked for systematic reviews and meta-analyses assessing the effects of EDs combined with alcohol using the words ‘energy drink and alcohol’, limiting the search for this kind of studies. Clinical trials published after 2 March 2017 were also screened.

All references published until 3 September 2021 were included.

## 3. Results

### 3.1. Mechanism of Action, Pharmacokinetics, and Adverse Effects

EDs have stimulant properties due to their caffeine content. They also contain other ingredients with pharmacological effects. Nevertheless, it should be taken into account that the quantities of the substances other than caffeine found in popular EDs could be lower than expected in order to produce either therapeutic benefits or adverse effects [14].

#### 3.1.1. Caffeine

Caffeine is a common ingredient in all EDs. A standard 250 mL ED contains 80 mg, which is equivalent to an average cup of coffee. The content of caffeine depends on the brand, ranging from 80 mg to 320 mg per unit [27]. The most concentrated EDs are usually called energy shots and can have up to 350 mg per unit. Caffeine may be derived from other ED ingredients such as guarana and yerba mate [14,28], and is also a component of some medicines [29].

Caffeine is a methylxanthine, in the same group as theophylline. It is a central nervous system (CNS) stimulant through its blockage of adenosine receptors, mainly A1 and A2A subtypes, thus competitively antagonizing their action. The absorption of adenosine by the CNS cells is one of the mechanisms that triggers sleep and sedation. Moreover, caffeine slightly increases the release of norepinephrine and dopamine, enhancing the neural activity of numerous brain areas. In addition, it exerts cardiovascular effects (chronotropic effect, hypotension, diuresis). Tachycardia can be explained by phosphodiesterase (PDE) 3 inhibition, A1 receptor antagonism, and catecholamine release. Hypotension is mediated by *β*2 adrenergic receptor-agonism while the diuretic effect is due to adenosine A receptor antagonism. Furthermore, caffeine acts as a PDE4 inhibitor causing bronchospasm, increased gastric secretions, headache, and vomiting. Bronchodilation can also be explained by A1 receptor antagonism, the catecholamine release is associated with headache and vomiting [13,30,31].

The main pharmacokinetic features of caffeine are rapid absorption when ingested orally, with a peak level in blood reached within 30 to 120 min, depending on the formulation [22,31,32]. It is metabolized into active methylxanthine metabolites through liver cytochrome P450 1A2 (CYP1A2) [31], including 3% to 10% into theophylline [32]. Caffeine metabolites are excreted in urine [32]. At lower dosages, caffeine follows first-order elimination kinetics. However, at higher concentrations, enzymatic saturation occurs, and metabolism converts it to zero-order kinetics. This may lead to accumulation and increased toxicity [33]. The elimination half-life of caffeine in adults ranges from 3 to 7 h [34] depending on CYP1A2 activity [31].

Caffeine poisoning symptoms have been extensively described according to the systems involved. Among the cardiovascular side effects, the following have been observed: increased blood pressure and heart rate [22,35], arrhythmias [35], coronary artery spasm [35,36], aortic dissection [35], acute ST-elevation myocardial infarction [35,36,37], stress-induced cardiomyopathy or Takotsubo cardiomyopathy [35,38]. Digestive and kidney disorders [39] include emesis [40], hepatitis [13], acute renal failure [13], rhabdomyolysis [22], and metabolic acidosis [22]. Obesity and hyperinsulinemia have been reported as metabolic adverse effects [22]. Neuropsychiatric adverse effects include epileptic seizures [13,14,24,32,37,40,41], reversible cerebral vasoconstriction [22], intracerebral haemorrhage [13,22], acute psychosis [13,22,37], and risk-taking behaviours [13] (for instance, hazardous driving with excessive speed and seat belt omission [22], sexual risk taking [22], and drug use [41]).

#### 3.1.2. Guarana

Guarana (*Paullinia cupana*) contains concentrated caffeine, up to 3.6–5.8% by weight (twice that found in coffee beans). Its mechanism of action, pharmacokinetics, and effects are therefore similar to those previously described for caffeine (see Section 3.1.1). Guarana intoxication produces water loss, gastric irritation, nausea, vomiting, metabolic acidosis, hyperglycaemia, ketosis, chest pain, syncope, tachydysrhythmia (a risk that is increased in the case of structural heart disease), shortness of breath, insomnia, nervousness, restlessness, tremors, anxiety, mania, seizures, and even sudden death [14,35,41].

#### 3.1.3. Yerba Mate

Yerba mate (*Ilex paraguariensis*) contains xanthines such as caffeine, theophylline, and theobromine. The dried leaves of the plant contain approximately 1–2% caffeine [42]. Effects described for caffeine can also be applied for this substance [19,28].

#### 3.1.4. Taurine

Taurine is a derivative of the amino acid cysteine naturally produced in the body [21]. It is sometimes erroneously classified as a CNS stimulant, although it has anxiolytic effects and interacts with the glutamatergic and GABAergic receptors [43]. In vitro studies have shown that taurine has inotropic effects on cardiac muscle similar to those of caffeine, and enhances caffeine-induced muscle contracture [36]. It also reduces blood pressure [21].

Taurine, once ingested and absorbed by active transport through the enterocytes, reaches the blood circulation and enters cells through the taurine transporter (TauT) [44]. Taurine, which does not seem to have liver metabolism, has urinary excretion [45]. In healthy volunteers, adverse effects appeared with high doses and consisted in muscle cramps [21]. Death has been documented only in combination with caffeine [14], or caffeine plus strenuous physical activity in physiologically predisposed individuals [36].

#### 3.1.5. Ginseng

Ginseng (*Panax ginseng*) is also a CNS/cardiac stimulant. Ginseng is used to relieve fatigue [46] and to treat hyperglycaemia because it has hypoglycaemic effects [21]. Intoxication leads to insomnia, headaches, vertigo, euphoria, mania, mastalgia, rashes, vaginal bleeding, amenorrhea, tachycardia, palpitations, hypertension, and oedema [34].

#### 3.1.6. *Ginkgo biloba*

*Ginkgo biloba* is one of the oldest living tree species. Most ginkgo products are made from the extract obtained from its fan-shaped leaves. It contains ginkgo-flavone glycosides, terpenes, lactones (ginkgolides), and 4’-O-methylpyridoxine (ginkgotoxin). Ginkgo-flavone glycosides have antioxidant effects while ginkgolides inhibit platelet-activating factor (PAF) facilitating blood flow [47].

#### 3.1.7. 5-Hydroxytryptophan

5-hydroxytryptophan is made from the seeds of the African plant *Griffonia simplicifolia*. It is a chemical precursor and metabolic intermediate in the biosynthesis of the neurotransmitter serotonin. It can be effective to treat depression, fibromyalgia, binge eating associated with obesity, chronic headaches, and insomnia [48].

#### 3.1.8. Bitter Orange

Bitter orange (*Citrus aurantium*) is also a stimulant. It contains an active ingredient called synephrine that is similar to ephedra [49]. Intoxication leads to myocardial infarction, hypertension, dysrhythmia, stroke, seizure, migraine, headache, and photosensitivity [14].

#### 3.1.9. Glucuronolactone

Glucuronolactone is a carbohydrate derived from glucose through its metabolism in the liver. It is transformed to L-ascorbic and xylulose (the enzyme involved is unknown) and excreted by the kidneys [50]. No reports of intoxication with this product have been found [19,34,51].

### 3.2. Interactions with Prescription Medication and Drugs of Abuse

As well as for pharmacological effects, caffeine is the primary ingredient of concern when considering ED interactions with other substances. EDs contain other ingredients that may interact with medicines and drugs of abuse, but none of these components, apart from caffeine and ginseng, have been reported as being a substrate, inducer, or inhibitor of CYP450, thus reducing the risk of pharmacokinetic interactions [42,52].

#### 3.2.1. Caffeine

Caffeine can interact with prescription and over-the-counter medication, herbs, and drugs of abuse. Such interactions could be pharmacokinetic (PK) or pharmacodynamic (PD) ones. The former appear when a change in concentrations may result in a change in effects while the latter are present when there is a change in effects [53].

Furthermore, individual pre-existing conditions or diseases, and inter-individual differences, mostly genetically determined, may condition caffeine metabolism in both directions (increase or reduction), contributing to a different individual “sensitivity” to the substance’s effects [31].

The main pharmacokinetic interactions of caffeine are with medication that is a substrate, inductor or inhibitor of CYP1A2 (see Table 1 [42,54]). While coffee is known to be a CYP1A2 inductor (polycyclic aromatic hydrocarbons are at least partially responsible), results of studies investigating caffeine CYP1A2 inducing effects are inconsistent [55].

CYP1A2 inducers may decrease caffeine concentrations while inhibitors can increase them, leading to adverse effects. In addition, caffeine interacts with legal and illegal drugs including tobacco, alcohol, and cannabis.

Tobacco smoke accelerates the metabolism of caffeine through CYP1A2 induction produced in liver microsomes by polycyclic hydrocarbons of tobacco [56]. Furthermore, an induction of CYP1A2 through cannabis smoking has also been reported, and therefore a reduction in caffeine concentrations is expected in both cases [25,57].

Additionally, acute alcohol ingestion inhibits caffeine metabolism through CYP1A2 [58,59,60].

Likewise, caffeine increases clozapine concentrations due to competitive inhibition of CYP1A2 [42,61]. It has also been reported that caffeine appears to raise the rate of absorption of paracetamol and levodopa with no clear changes in bioavailability [42].

Caffeine augments lithium excretion. A reduction in its consumption can therefore increase lithium concentrations and adverse effects [25].

Coadministration with caffeine may raise the serum concentrations of theophylline. The proposed mechanism involves competitive inhibition of theophylline metabolism via CYP450 1A2, as well as metabolic conversion of caffeine to theophylline in vivo, and saturation of theophylline metabolism at higher serum concentrations [25].

The PD interactions of caffeine can be classified as synergistic, additive, and antagonistic in nature. When the overall effect caused by a drug combination is the sum of the pharmacological effects of each individual drug an additive interaction occurs. Synergy is produced when the overall effect of the drug combination is greater than additive, and antagonism takes place when the drug combination effect is less than additive [42].

Synergic or additive effects of caffeine with other psychostimulants such as cocaine, amphetamines, 3,4-methylenedioxymethamphetamine (MDMA), and cathinones are expected. Tachycardic and hypertensive effects of sympathomimetics may be enhanced by caffeine, and myocardial ischemia can appear with concurrent use [10].

Caffeine increases postprandial hyperglycaemia and reduces insulin sensitivity, and may decrease the effectiveness of diabetes medication (insulin and oral hypoglycaemic agents) [25,62]. Caffeine also reduces methotrexate efficacy possibly due to its antagonistic effects on adenosine receptors. Adenosine accumulation results in anti-inflammatory effects [25].

Furthermore, caffeine might decrease blood clotting. The ingestion of caffeine jointly with medication that reduces clotting might increase the chances of bruising and bleeding. Some of these include aspirin, clopidogrel, diclofenac, ibuprofen, naproxen, dalteparin, enoxaparin, heparin, and warfarin. In addition, caffeine modestly raises the bioavailability, rate of absorption, and plasma levels of aspirin [42].

On the other hand, caffeine partially antagonizes the effects of sedative drugs such as benzodiazepines [63,64,65], Z-drugs [64,66], and alcohol [67,68,69].

During alcohol intoxication, caffeine antagonizes the somnogenic effects of alcohol by blocking the adenosine A1 receptors, while the anxiolytic effects of alcohol could compensate for the anxiogenic effects of caffeine [67].

Additionally, caffeine blocks peripheral pro-nociceptive adenosine signalling, improves analgesic absorption through lower gastric pH and changes perception of pain. It has been shown to enhance analgesic effects of paracetamol/acetaminophen, acetylsalicylic acid/aspirin, and ibuprofen [70].

#### 3.2.2. Guarana and Yerba Mate

As guarana and yerba mate contain caffeine, interactions for caffeine can be applied to them [19,21,28].

#### 3.2.3. Taurine, Ginseng and Bitter Orange

Taurine, ginseng, and bitter orange may interact with other stimulants, or have antagonistic effects with sedative drugs, as they are considered stimulants [14]. Taurine may increase the physiologic actions of caffeine in cardiovascular system [71]. Taurine with caffeine leads to an increase in blood pressure instead of a decrease [71]. There is, however, insufficient evidence to attribute such effects to taurine intake [14] or to suggest an unsafe level of consumption [36].

Coadministration of ginseng with antidiabetic drugs (insulin and oral hypoglycaemic agents) may increase the risk of hypoglycaemia (unknown mechanism) [25,42,72]. Caution should be exercised when taking ginseng with anticoagulant or antiplatelet drugs as ginsenosides can decrease platelet aggregation and lower warfarin plasma concentrations (unknown mechanism) [25,72,73]. Furthermore, ginsenosides have been reported to decrease plasma alcohol concentrations due to the induction of the microsomal ethanol oxidizing system or a delay in gastric emptying [74,75]. Ginseng inhibits CYP3A4, responsible for imatinib metabolism. Their combination can cause liver toxicity [52]. Furthermore, ginseng may falsely increase digoxin levels and have additive effects with corticosteroids and oestrogens [76].

#### 3.2.4. *Ginkgo biloba*

Increased risk of bleeding is possible when *Ginkgo biloba* is combined with medication that has antiplatelet action (e.g., warfarin, acenocumarol, acetylsalicylic acid, clopidogrel, dabigatran, rivaroxaban, ibuprofen). 4′-*O*-methylpyridoxine competes with vitamin B6, which causes an indirect inhibition of glutamate decarboxylase and subsequent decrease in the formation of gamma-aminobutyric acid (GABA) in the brain. A theoretical increased risk of seizure is expected when combined with other agents that can lower the seizure threshold such as selective serotonin reuptake inhibitors (SSRI antidepressants and anorectics), monoamine oxidase inhibitors, neuroleptic agents, CNS stimulants, opioids, tricyclic antidepressants, other tricyclic compounds (e.g., cyclobenzaprine, phenothiazines), carbapenems, cholinergic agents, fluoroquinolones, interferons, chloroquine, mefloquine, lindane, and theophylline [25,47,72,73].

#### 3.2.5. 5-Hydroxytryptophan

5-hydroxytryptophan can interact with antidepressants, or other agents with serotonergic activity, and augment the risk of serotoninergic syndrome [25].

#### 3.2.6. Other Ingredients

Interactions between B vitamins contained in EDs and medicines are not expected. Nevertheless, the high carbohydrate load associated with ED ingestion should be taken into account in diabetic patients in order to appropriately adjust their insulin regimens [77]. No reports of interaction with glucuronolactone have been found.

#### 3.2.7. Interactions with Energy Drinks

The potential interactions of EDs with medicines and drugs of abuse include those previously reported for individual components.

The interaction of EDs with alcohol deserves special attention because this mix is trendy in adolescents and young people. In all, 16% of students reported consumption of alcohol mixed with ED (AmED) [11]. Reasons include reducing the unpleasant taste of alcohol, or counteracting alcohol sedative effects thus allowing longer drinking sessions.

The counterpart of such a combination could be a predisposition to adopt risky behaviours whilst under its influence, or the predisposition to alcohol use disorders. It has been reported that the caffeine content of EDs enhances the rewarding effects of alcohol [67]. The combination of alcohol with EDs increases the risk of cardiovascular complications as both produce tachycardia [21].

Several experimental studies have been conducted to assess whether there is an acute interaction between alcohol and EDs on a number of outcomes (intoxication, subjective outcomes, psychomotor and cognitive performance). Evidence has been summarized in at least 7 different systematic reviews included in Table 2 [78,79,80,81,82,83,84]. In summary, in experimental conditions, EDs combined with alcohol did not reduce drunkenness [78,82,84]. They did, however, reduce sedation and partially improve alcohol-induced impairment of cognitive and psychomotor performance [79,80,84]. Effects on alcohol consumption or engagement in risk-taking behaviours are not clear as mixed results have been found [80,81,82].

Since the last meta-analysis [82] another study has been published [78] assessing the effects of AmED on attentional and working memory. Like in previous studies, mutual antagonism was not evident in all tasks and depended on alcohol and ED dose, and the blood alcohol limb [85].

The most recent studies [86,87] assessed the effects of AmED on driving-related skills, and showed that EDs increase willingness to drive while drunk, but did not, or only partially counteract alcohol effects on objective performance outcomes. An increase in alcohol and caffeine plasma concentrations was also observed with AmED in comparison with alcohol or ED alone [86]. EDs impair judgement of alcohol perceived intoxication in observational studies [88]. Altogether these results suggest higher risk while driving under their influence.

### 3.3. Intoxications with EDs and Management

Published cases of intoxication with EDs have been screened to identify the substances most concomitantly consumed and the reported effects. The main characteristics of each case report are summarized in Table 3 for combination with alcohol [89,90,91,92,93] and Table 4 for combination with other drugs of abuse [1,16,33,85,88,94,95,96,97].

Intake of more than three cans of EDs, or less in poor tolerance individuals, can lead to caffeine intoxication [14].

ED intoxication comprises a wide range of symptoms. Intoxicated patients more often will be adolescents or young people who party all night. The patient may present palpitations, chest pain, agitation, headache, insomnia, tremors, seizures, nausea and vomiting, with or without increased diuresis.

Faced with these symptoms, the emergency clinician should ask the patient about the consumption of EDs, as well as other stimulant substances. If the patient has cardiac symptomatology, reports ingestion of several EDs in a short period of time or the equivalent to more than >500 mg of caffeine/day [19,31,34] in adults (>3 mg/kg body weight per day in children) [98] an electrocardiogram should be performed. Blood pressure and heart rate monitoring is recommended till caffeine concentrations are reduced to non-toxic levels (according to half-life the time needed to eliminate all caffeine is 15–35 h).

Likewise, it is recommended to inquire about the concomitant use of other drugs and any medication, since both caffeine and other components of EDs could interact with them, as mentioned before.

If the patient in the emergency room has been involved in a traffic accident, has aggressive behaviour or sexual alterations, the concomitant use of alcohol with EDs must be ruled out [12,99,100]. If the patient reports consumption of medicines, other drugs or has a positive urine screen test, the pertinent algorithm for each type of intoxication must be followed [101].

Regarding medication it is important to keep in mind that EDs can increase the effects of drugs such as clozapine, paracetamol/acetaminophen, lithium and theophylline; and reduce the effects of diabetes medication, methotrexate, sedative drugs and decrease blood clotting (might increase the effects of drugs that reduce clotting too) [25,42,61,62,63,64,65,66,67,68,69].

Treatment of caffeine intoxication is symptomatic and supportive as there is no specific antidote. If ingestion has occurred within one hour of the emergency visit, a single dose of activated charcoal can be administered. Nausea and vomiting can be treated with ondansetron. Benzodiazepines are indicated for CNS excitation (tremors, anxiety, and agitation). If seizures are refractory barbiturics and propofol can be considered [13].

Medical therapy for supraventricular tachycardia and ventricular dysrhythmias is available in specific guidelines [102].

### 3.4. Caffeine/ED Substance Use Disorder

The existence of a caffeine use disorder is still a topic of debate. It is not yet clear to what extent it is a clinically significant disorder, and for this reason not included in the fifth edition of the Diagnostic and Statistical Manual of Mental Disorders (DSM-5) [103]. In parallelism, there is thus insufficient evidence to talk about the existence of an ED use disorder.

DSM-5 includes the following disorders related to caffeine: caffeine intoxication, caffeine withdrawal, other caffeine-induced disorders (e.g., anxiety and sleep disorders, as caffeine can aggravate symptoms of anxiety, panic disorder, and insomnia), and unspecified caffeine-related disorder [103,104].

Caffeine intoxication diagnosis is assigned to individuals who experience symptoms including restlessness, nervousness, insomnia, or digestive issues after consuming a high dose. Caffeine intoxication meets the following criteria: recent caffeine use, usually in excess of 250 mg, and five or more symptoms that develop shortly thereafter, such as restlessness, nervousness, insomnia, gastrointestinal disturbance, and tachycardia [103,104].

Caffeine withdrawal disorder is characterized by prolonged daily use of caffeine and three or more withdrawal symptoms occurring within 24 h of abrupt cessation/reduction in intake. These include headache, marked fatigue or drowsiness, dysphoric mood/depressed mood/irritability, difficulty in concentration, and flu-like symptoms. Symptoms usually begin 12–24 h after the last caffeine dose and peak after 1–2 days of abstinence with spontaneous amelioration [103,104].

Unspecified caffeine-related disorder applies to presentations in which symptoms characteristic of a caffeine-related disorder causing clinically significant distress or impairment in social, occupational, or other major areas of functioning predominate. Nevertheless, they do not meet the full criteria for any specific caffeine-related disorder, or any of the disorders in the substance-related/addictive disorder diagnostic class [103,104].

## 4. Discussion

Based on our review, we have made several suggestions on the attitude that the physician should have towards the consumption of EDs. Patients should be informed about possible short- and medium-term adverse effects of ED consumption, as well as the possibility of interactions with other substances. In addition, it has been proposed what the management should be in case that an intoxicated patient needs to be treated in the emergencFy room (based on the management of caffeine intoxication).

The consumption of EDs with medicines and drugs of abuse is, in general terms, not recommended as potential interactions and adverse effects can appear, mainly due to the high ED caffeine content. Patients exposed to polypharmacy (e.g., patients living with diabetes) are those at higher risk of drug interactions and should refrain from the consumption of these products [105]. EDs consumption could be replaced with natural sources of caffeine such as coffee or tea, which also may have cardiovascular benefits [106].

In order to reduce the risk of ED interactions, it is advisable for physicians to ask about EDs consumption and consult the product information of the prescribed medication (the leaflet in the case of patients) [26]. They should look for interactions with caffeine or other psychostimulants that would include EDs. Online databases of interactions may also be helpful [25] as it could be to check a review manuscript like this one.

Children and adolescents are at the greatest risk of suffering the possibly deleterious effects of acute and regular ED consumption. In fact, the information available is insufficient to establish a safe caffeine intake, and the level of safety concern set at 3 mg/kg body weight per day is extrapolated from adult data [98]. Several studies have linked prolonged consumption of EDs with a more frequent consumption of drugs of abuse, such as cocaine and alcohol. It has been hypothesized that the consumption of substances with the greatest addictive potential may be favoured by ED intake in adolescence [16,22,97]. ED consumption also has been linked to other risky behaviours in young people, such as sexual risk-taking, fighting, and seat belt omission [107]. Because of these reasons, we are convinced of the importance of informing adolescents and young adults and their families of these possible risks. There is as yet insufficient evidence regarding the safety of regular consumption of these drinks [108]. Nevertheless, it should be taken into account that concomitant use of caffeine and sugary soft drinks in children and adolescents may result in poor dietary habits across the lifespan, and increase the risk of obesity and dental caries [109,110]. In sedentary individuals, insulin resistance may explain the deleterious effects of EDs [111].

In our opinion, knowledge concerning the acute and long-term risks of consuming such beverages should be improved with educational programs at schools focused on children and their parents. In addition, health professionals should screen ED consumption in young people, as is done for other dietary habits and drugs, and provide advice to reduce associated risks. It could be beneficial to add ED consumption to ongoing youth health monitoring programs [24,107] and in emergency poisoning algorithms.

## 5. Conclusions

Taking into account the broad consumption of EDs throughout the world, the scarce information about their potential interactions with medicines and drugs of abuse is of concern. EDs can interact with substrates, inductors or inhibitors of CYP1A2, psychostimulants, alcohol and other depressant drugs.

The greatest consumers of EDs are young people. They also tend to combine EDs with alcohol and consume drugs of abuse, thus presenting a higher risk of interactions. Healthcare professionals should provide information about such potential health and behavioural risks in order to raise awareness among adolescents and their parents [107].

Acute effects in various body systems have been reported in ED intoxications including cardiac, digestive, psychiatric, and neurological conditions. Even fatal cardiac events have been reported [36]. Regarding long-term effects, both metabolic issues [109] and greater susceptibility to the consumption of drugs of abuse have been described [112]. However, data on long-term safety are scarce.

To conclude, during the medical interview, questions should be raised regarding the consumption of EDs, in addition to other substances, in order to reduce adverse effects and avoid potential drug interactions that could have fatal consequences.

## Figures and Tables

**Table 1:** List of CYP1A2 substrates, inducers and inhibitors.

Substrates		Inducers	Inhibitors	
agomelatine alosetron amitriptyline asenapine caffeine chlorpromazine clomipramine clopidogrel clozapine cyclobenzaprine dacarbazine domperidone duloxetine ergotamine estradiol flutamide fluvoxamine frovatriptan guanabenz haloperidol imipramine lidocaine melatonin mirtazapine nabumetone	nacetamoline nacetamoline naproxen ondansetron olanzapine oxytamine paracetamol phenacetin pirfenidone pomalidomide propafenone propranolol ramelteon ramosetron riluzole ropivacaine ropirinole R-warfarin tacrine tasimelteon theophylline tizanidine triamterene verapamil zolmitriptan	antipyrine carbamazepine coffee insulin lansoprazole nafcillin nelfinavir omeprazole phenobarbital phenytoin pentobarbital polycyclic hydrocarbons (tobacco smoke) primaquine rifampin ritonavir secobarbital sulfinpyrazone teriflunomide	acyclovir allopurinol amiodarone cimetidine clarithromycin disulfiram duloxetine efavirenz enoxacin erythromycin famotidine fluoroquinolones fluvoxamine grapefruit juice isoniazid ketoconazole methoxsalen mexiletine oral contraceptives peginterferon-alfa-2a piperine propafenone rhytomycin ticlopidine tolfenamic acid	troleandomycin rofecoxib verapamil zafirlukast zileuton

**Table 2:** Published systematic reviews of the effects of AmED.

Recreational Drug	Reference	Type of Study	Number of Studies	Dose of EDs	Dose of Alcohol	Outcomes	Results	Type of Interaction
AmED	McKetin et al., 2015	Systematic review	62 studies	80 mg of caffeine	2–7 drinks	Effects on alcohol consumption, intoxication, alcohol-induced impairment, breath alcohol concentration	AmED reduces alcohol-induced impairment on some, but not all aspects of complex tasks	PD
	Verster et al., 2018	Systematic review and meta-analysis	80 original articles	NA	NA	Alcohol consumption Subjective intoxication Risk-taking behaviour	No increase in alcohol consumption or risk-taking behaviours No effect on subjective intoxication	PD
	Benson et al., 2014	Systematic review and meta-analysis	9 studies (4 with ED)	1.2 mg/kg–5.5 mg/kg of caffeine	0.29 g/kg–1.068 g/kg	Subjective intoxication	Caffeine had no effect on the judgement of subjective alcohol intoxication	PD
	Lalanne et al., 2017	Systematic review	12 studies	2–4 mg/kg of caffeine or 80 mg (an ED can)	0.6 g/kg–1.068 g/kg	Dual task interference and motor coordination ability to drive, alcohol-induced subjective effects, risk of developing addictive behaviours	EDs counteract some cognitive deficits and adverse effects of alcohol, but not in complex tasks. AmED increases the risk of alcohol use disorder	PD
	Peacock et al., 2014	Systematic review	19 studies	3.57 mg/kg–80 mg of caffeine	0.65 mg/kg	Physiological, psychological, cognitive and psychomotor outcomes, hazardous drinking practices, risk-taking behaviour	AmED increases stimulation and reduces sedation. AmED consumers report more hazardous alcohol consumption patterns and greater engagement in risk-taking behaviour	PD
AmED	Roemer et al., 2017	Systematic review	13 studies	NA	NA	Drinking behaviours, impulsivity or risk-taking propensity, self-reported injury outcomes.	Association between AmED and increased risk of injury	PD
	Verster et al., 2016	Systematic review and meta-analysis	14 studies	NA	NA	Consumption pattern	AmED has no significant impact on total alcohol consumption on a typical single drinking occasion	PD

ED: energy drink; NA: not available; PD: pharmacodynamic; PK: pharmacokinetic.

**Table 3:** Intoxications from AmED, and surveys reporting AmED use.

Recreational Drug	References	Type of Study	Number of Studies	Dose of EDs	Dose of Alcohol	Outcomes	Results	Type of Interaction
AmED	Snipes et al., 2013	Online survey	704 undergraduate students 84.7% alcohol 19.4% AmED	NA	NA	Behavioural disturbance, Sexual risk behaviour Unprotected sex Alcohol dependence Binge drinking Potential for sexually transmitted infections	AmED consumers were more likely to use marijuana, ecstasy, and cocaine	PD
	Bonar et al, 2015	Emergency department medical records	2414 cases (range 14–20 years old) 36% AmED	NA	NA	Feeling jittery, restless, on edge, or nervous, insomnia, increased alertness, stomach pain or irritation, feeling irritable, irregular heartbeat, headaches, muscle twitching, had to go to doctor, risk behaviours, alcohol use severity	Participants reported medical consequences of combined use and higher rates of other risk behaviours (sexual, substance use and driving-related risk behaviour)	PD
	Matuszkiewicz et al., 2015	Case report	1 subject	NA	NA	GCS 8 points. Somnolence, restless, seizures, respiratory insufficiency	NA	PD
	Snipes et al., 2015	Online survey	757 undergraduate college students 11.6% AmED	NA	NA	Impulsivity Alcohol dependence	AmED consumption associated with problematic alcohol consumption	PD
	Caviness et al., 2017	Phone interview	481 young adults (18–25 years old) 31.2% AmED	NA	Alcohol use 7, 9 ± 5.5 days per month	Binge drinking Alcohol use disorder	Higher binge drinking, negative consequences and rates of alcohol use disorder among AmED consumers	PD

ED: energy drink; GCS: Glasgow Coma Scale; NA: not available; PD: pharmacodynamic; PK: pharmacokinetic.

**Table 4:** Intoxications and surveys reporting use of EDs with other drugs.

Recreational Drug	References	Type of Study	Number of Subjects	Dose of EDs	Dose of Drug	Clinics/Outcomes	Results	Type of Interaction
MDMA	Israelit et al., 2012	Case report	1 subject, 24 years	20 cans of XL 2000 mg of caffeine	NA	Chest pain, nausea, vomiting, acute myocardial infarction	Ventricular fibrillation and death	PD
Cannabis Tobacco Binge drinking	Hamilton et al., 2013	Survey of drug use	4342 students (grades 7–12)	NA	NA	Sensation seeking Use of other drugs Binge drinking	EDs consumption was highly associated with tobacco, cannabis, non-medicinal use of prescription drugs, and binge drinking	PD
Cannabis Tobacco Binge drinking	Ali et al., 2014	Systematic review of case reports	43 reports	1–24 cans	NA	Chest pain Seizures Psychomotor agitation	EDs use is associated with substance abuse leading to increased risky behaviours as well as injuries	PD
Alcohol Marijuana Cocaine Meth	Nordt et al., 2017	Survey in emergency department	270 subjects (13–17 years) 192 ED (24% + ethanol illicit drugs)	NA or	NA	Insomnia, jittery, palpitations, abdominal pain, nausea, vomiting, or diarrhoea, headache, chest pain, dyspnoea and seizures	40% reported an adverse event	PD
Ecstasy Polydrug use	Pennay et al., 2017	Survey drug use	7028 individuals22.6% ED ecstasy 4.0 cannabis 2.9% meth 2.6%, cocaine 1.6% polydrug 2%	NA	NA	No description	ED correlated with ecstasy use (OR 1.81) and polydrug use (OR 1.53). The latter more involved in physical aggression and accidents or injuries	PD
Alcohol Ecstasy	Peacock et al., 2016	Survey of regular ecstasy users	693 individuals (>16 years old) 77% ED + alcohol (70%) ecstasy(57%)	3 EDs (range 1–30) on their last drinking occasion	NA	Headache, heart palpitations, nausea, vomiting, on edge, heart burn, stressed out	50% of AmED and 25% of ED + ecstasy consumers reported at least one negative adverse outcome	PD
Alcohol Illegal stimulants	Kaestle et al., 2017	Brief interview	4628 participants 6% > 3 ED 8.2% stimulants	NA	NA	Subjective intoxication Blood alcohol concentration (BAC)	Co-consumption of ED/stimulant drugs with alcohol impairs intoxication judgement	PD
Alcohol Stimulants	Housman et al., 2016	Survey	1304 students (grade 12). 44% AmED (8.3% non-medical use of Ritalin and 20.8% of Adderall)	NA	NA	Anxiety, irritability, agitation, insomnia, increased systolic blood pressure, cardiac abnormalities, seizures and tachycardia	Greater frequency of AmED use was also associated with greater frequency of Ritalin and Adderall use	PD
Alcohol Stimulants Cannabis	Mcketin et al., 2014	Online survey alcohol users	1994 cases (18–30 years old) 63% alcohol and 21% ED (last Saturday) 497 ecstasy/688 cannabis past year	NA	NA	Drug intoxication Binge drinking Alcohol use disorder	A strong association between stimulant intoxication and excessive alcohol consumption	PD

ED: energy drink; MDMA: 3, 4-methylenedioxymethamphetamine; meth: methamphetamine; NA: not available; PD: pharmacodynamic; PK: pharmacokinetic.

## Data Availability

Not applicable.
